# Supplementary material for: Long-term non-invasive drug treatments in adult zebrafish that lead to melanoma drug resistance
Source: Dis Model Mech. 2022 May 9;15(5):dmm049401. doi: 10.1242/dmm.049401 (PMC9118090; doi:10.1242/dmm.049401)
Supplement: Supplementary information [file dmm-15-049401-s1.pdf]

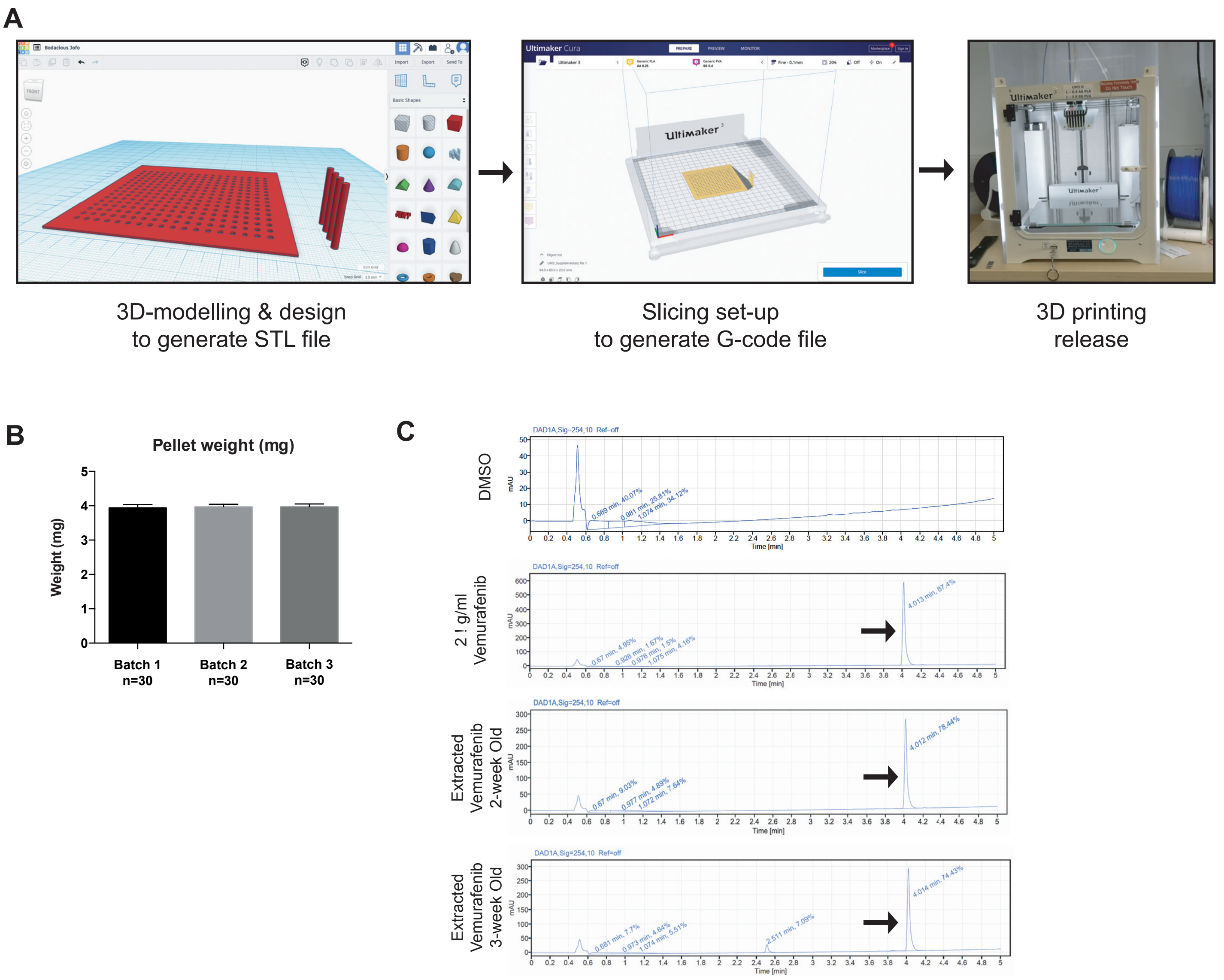

D

	Pure Vemurafenib in DMSO			Vemurafenib Extracted from 2 Pellets by DMSO									
Sample	2.5 µg/ml	2 µg/ml	1 µg/ml	fresh		week 0.5		week 1		week 2		week 3	
Peak Area	1524.4	1154.5	631.7	543.1	622.5	499.3	427.7	543.4	430.4	563	527.4	293.2	350.6
Height	775.9	589.6	322.3	277.2	316.8	254.6	218.8	278.4	219.5	287.2	269	149.3	178.7
Percentage	90.24	87.4	80.8	92.6	80.4	77.74	73.39	78.44	73.62	74.43	74.56	66.27	69.46

Disease Models & Mechanisms • Supplementary information

Fig. S1. Drug pellet manufacturing and batch consistency validation

A. The pipeline concept of 3D-design and printing the pressing mould. Briefly, the 3D-design software (e.g. Tinkercad) provides a millimeter-based sketching interface to allow precise modelling of the mould and generates the STL file with the 3D-surface geometry information stored. Next, the slicing software (e.g. Ultimaker Cura) generates the customised G-code file with instructions for printing materials and nozzle settings. The G-code file is ready-for-print on the selected 3D-printers.

B. The weight of drug pellets produced from three independent batches. Drug-pellets weighed 4.0 mg ± 0.2 mg. (mean±s.d., n=30, one-way ANOVA, p=0.38, non-significant).

C. Representative chromatograms for DMSO, vemurafenib dissolved in DMSO, and vemurafenib drug pellets dissolved in DMSO after 2-week or 3-week storage at -80°C. The signature peak of vemurafenib is highlighted by black arrows (molecular weight 490 g/mol measured by HPLC and reported as 489.92 g/mol by manufacturer).

D. Chromatogram peak measurement results of vemurafenib dissolved in DMSO and vemurafenib drug pellets dissolved in DMSO (freshly prepared), or after 1-, 2- or 3-week storage at -80°C.

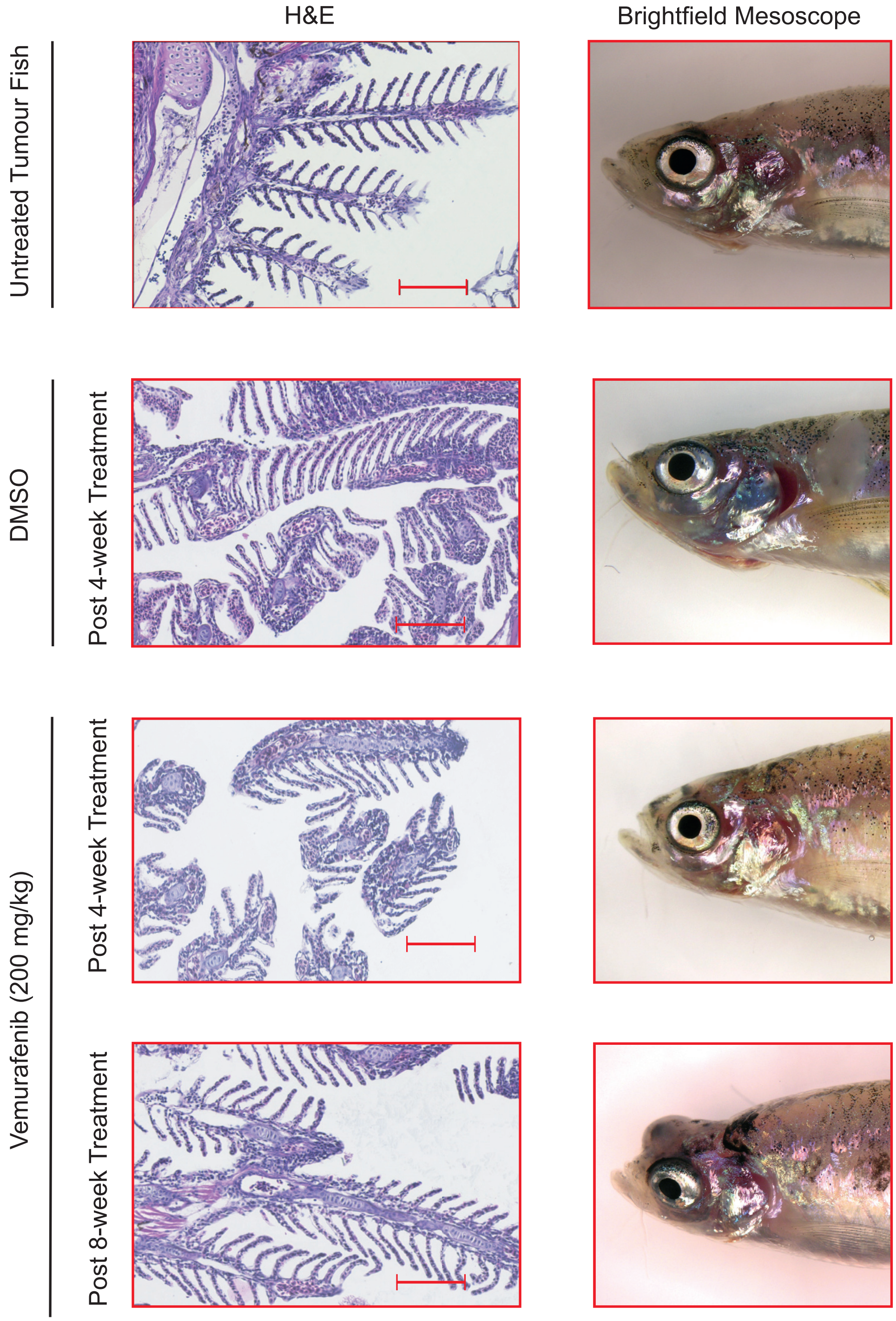

Disease Models & Mechanisms • Supplementary information

Fig. S2. Gill health not affected after drug pellet treatments.

Representative H&E staining gill tissues of untreated BRAF^{V600E} zebrafish versus DMSO or vemurafenib drug-pellets treated BRAF^{V600E} zebrafish. Scale bar = 100 μm. The brightfield mesoscope fish images featuring the gill area matching the H&E sections are shown on the right. DMSO treated fishes were not followed beyond 4-week of treatment because of the increased tumour burden and welfare implication for the animal.

For comparison to non-tumour wildtype fish gill histology, virtual slide resources are available from the Zebrafish Information Network (ZFIN); under the project Bio-Atlas (http://zfatlas.psu.edu/). An example slide: http://bio-atlas.psu.edu/view.php?s=182&atlas=16.

Table S1. Reagents and resources used in this study

REAGENT or RESOURCE	SOURCE	IDENTIFIER
**Antibodies and Chemicals**		
Phospho-p44/42 MAPK (Erk1/2) (Thr202/Tyr204) Antibody	Cell Signaling	Cat No: #9101
p44/42 MAPK (Erk1/2) Antibody	Cell Signaling	Cat No: #9102
Goat anti-Rabbit IgG (H+L) Highly Cross-Adsorbed Secondary Antibody Alexa Fluor® 488 conjugate	Thermo Fisher Scientific (Life Technologies)	Cat No: A-11034
Vemurafenib (PLX4032)	SelleckChem	Cat No: S1267
Dimethyl sulfoxide (DMSO)	SigmaAldrich	Cat No: 67-68-5
**Commercial Food Ingredient**		
ZM Flakes	ZM Fish Food and Equipment	N/A
ZM Medium Premium Granular	ZM Fish Food and Equipment	N/A
ZM Small Granular	ZM Fish Food and Equipment	N/A
Hikari Micro Pellets	Kyorin Food Industries, Ltd.	N/A
Cooks' Ingredients Agar Agar	Waitrose & Partners	N/A
Dr Oetker Gelatine	Dr Oetker KG	N/A
**Experimental Models: Organisms/Strains**		
Tg(mitfa:BRAFV^{600E}), p53M^{214}K	Patton et al. (2005)	ZFIN Cat #: ZDB-TGCONSTRCT-070117-106, ZDB-ALT-050428-2
**Software and Algorithms**		
TinkerCAD	https://www.tinkercad.com/	N/A
Ultimake Cura	https://ultimaker.com/software/ultimaker-cura	RRID:SCR_018898
GraphPad Prism	http://www.graphpad.com/	RRID:SCR_002798
Fiji 1.0	http://fiji.sc	RRID:SCR_002285

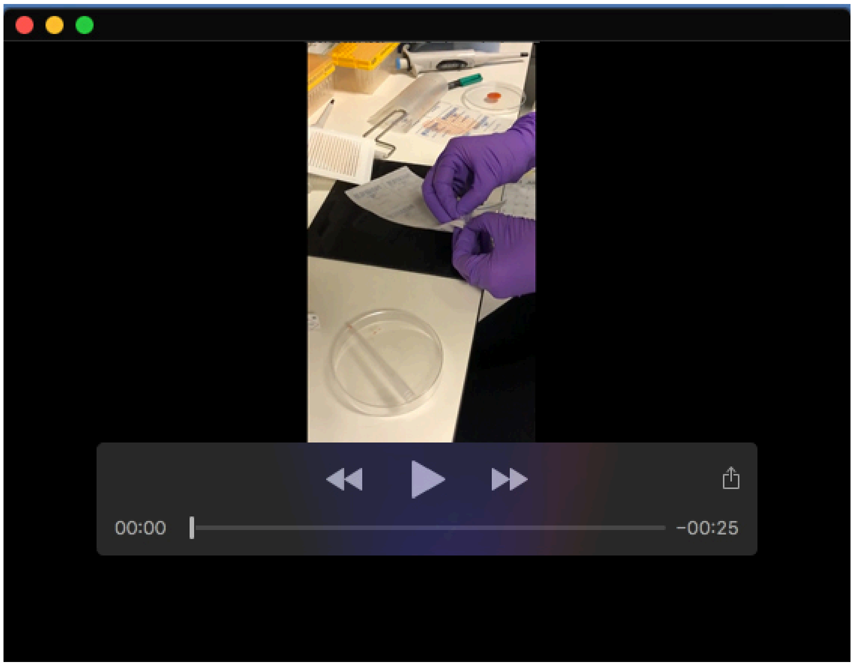

Movie 1. Preparation of drug pellets.
Drug supplemented food-agar mixture paste was cooled in a petri-dish, and once partially congealed, the jelly-like paste was transferred onto the 3D-printed mould and pressed into pellets between the parafilm sheet and backing paper.

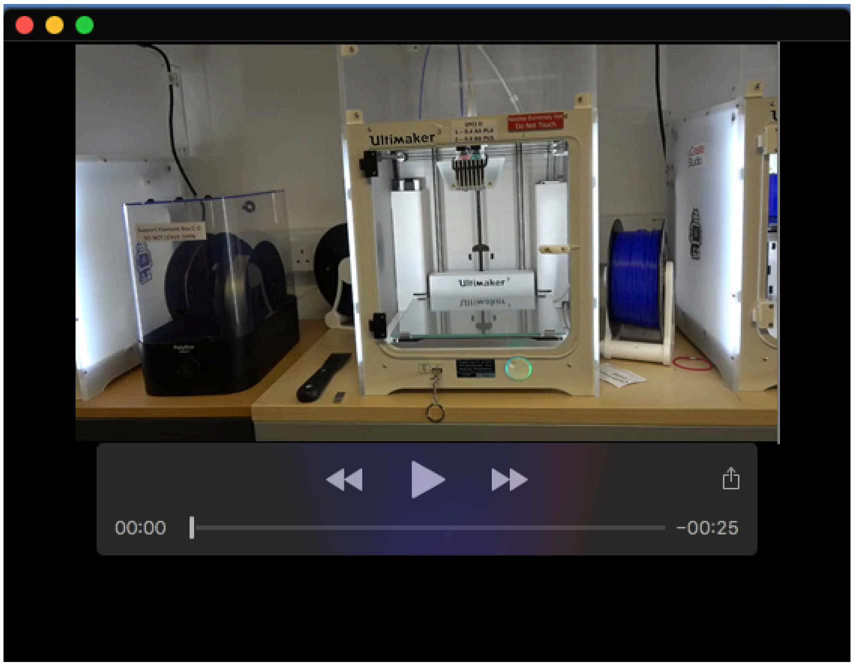

Movie 2. The design and 3D-printing the drug-pressing mould.
The 3D-modelling and design of the drug-pressing mould was carried out on the open-source website-based software Tinkercad to generate an STL file, which contains the three-dimensional surface geometry information of the designed object **(Dataset 1).** The STL file was then imported into a slicing modelling software to choose the customised parameters for paired 3D-printers. In our case, we used the software Ultimaker Cura for slicing and generated a G-code file ready for the 3D-printer Ultimaker 3 to print out the object, with the nozzle set-up as AA 0.25 and to use the generic PLA as the printing material. The printing process itself is highly automated and only requires the user to select the file to print. After the printing, the PLA material must cool and become firm before retrieving the object. Occasional disfigurations might occur during the process of printing which is largely due to residual grease fingerprint left on the bottom of the printing surface due to human touch (using 70% ethanol wiping clean the surface will help reduce such incidents).

Disease Models & Mechanisms • Supplementary information

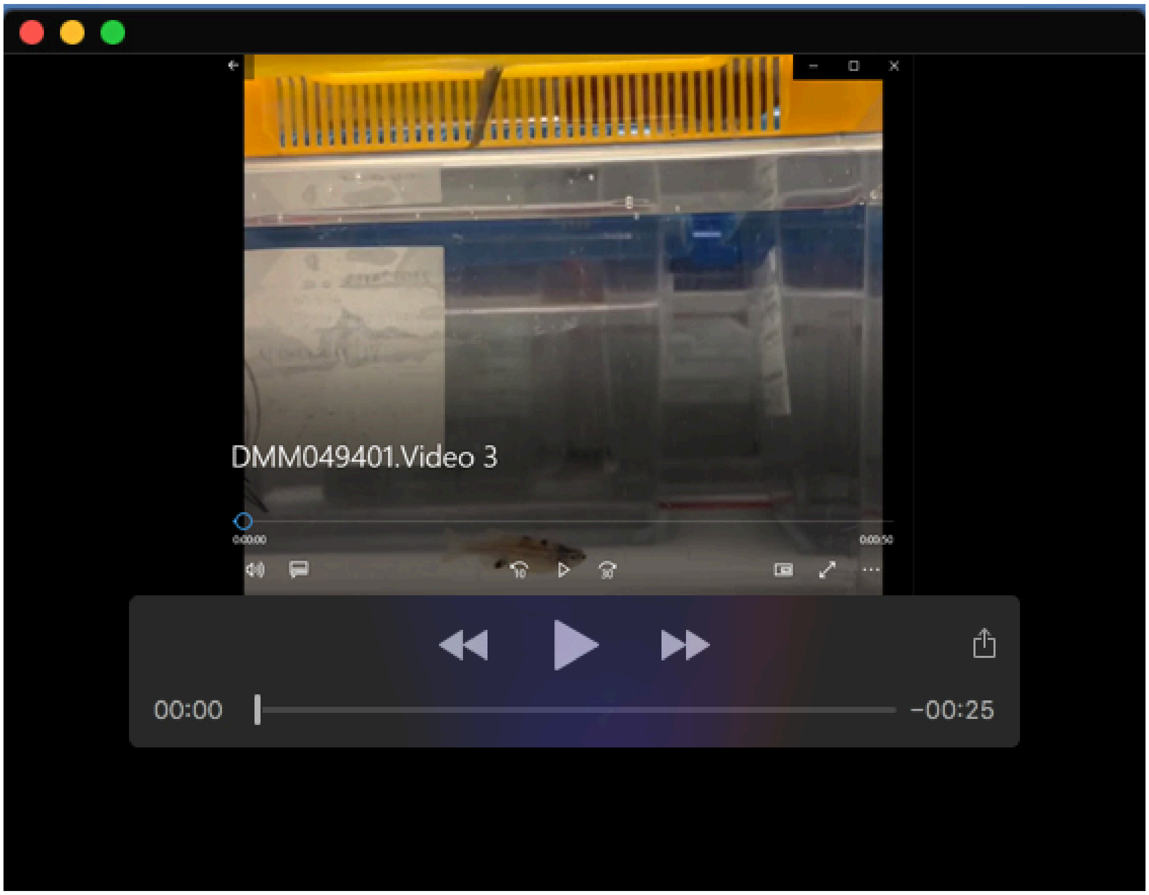

Movie 3. Free-feeding adult zebrafish consume drug pellets.
Single-housed adult zebrafish were fed once daily with artemia during the day and given drug-pellets in the late afternoon. The video shows how zebrafish actively sought for and consumed the drug pellet voluntarily without any handling.

Dataset 1. Design of the drug-pressing mould

Click here to download Dataset 1
